# The relationship between public stigma, self-stigma, and non-suicidal self-injury in outpatient adolescents: a cross-sectional study

**DOI:** 10.3389/fpsyt.2025.1681654

**Published:** 2025-11-03

**Authors:** Qing Guo, Jiayuan Wang, Ruofei Wang, Ruofan Wang, Zikang Liu

**Affiliations:** ^1^ Department of Psychiatry, The Affiliated Hospital of Northwest University Xi’an No.3 Hospital, Xian, China; ^2^ Department of Psychiatry, First Affiliated Hospital of Xi’an Jiaotong University, Xian, China; ^3^ Department of Psychology, Chengde Medical University, Chengde, China; ^4^ Beijing Huilongguan Hospital, Beijing, China; ^5^ Faculty of Psychology, Tianjin Normal University, Tianjin, China

**Keywords:** adolescents, non-suicidal self-injury, self-stigma, public stigma, personality traits

## Abstract

**Introduction:**

Stigma has a profound impact on the mental health of adolescents. However, empirical evidence on the association between stigma and non-suicidal self-injury among outpatient adolescents remains limited. This study aims to investigate the levels of stigma and self-injury among outpatient adolescents and to explore the relationship between stigma and self-injury.

**Methods:**

A total of 130 adolescents aged 12 to 18 who met the DSM-5 diagnostic criteria for non-suicidal self-injury and visited the psychiatric outpatient clinic between February and October 2024 were recruited for the study. The assessment included the adolescent self-harm questionnaire, the self-stigma of psychiatric disorders scale, the perceived devaluation-discrimination scale, and the Eysenck personality questionnaire-junior version. Statistical analyses were conducted using SPSS 26.0 and Rx64 4.0.3, with regression analyses employed to examine the effects of stigma and personality traits on the severity and frequency of self-injury among outpatient adolescents.

**Results:**

(1) Among the 130 outpatient adolescents, the mean score for self-stigma was 75.13 ± 14.28, and the mean score for public stigma was 32.35 ± 6.25. A total of 63 adolescents (48.50%) exhibited a high severity of self-injurious behavior. (2) The proportion of adolescents with high severity of self-injury was significantly higher among those with divorced parents (χ²=5.898, P = 0.015) and those with a family history of psychiatric disorders (χ²=9.922, P = 0.003). Moreover, adolescents with a family history of psychiatric disorders had a higher frequency of self-injury compared to those without such a history (t=-2.637, P = 0.009). (3) The frequency of self-injury among outpatient adolescents was positively correlated with self-stigma (r=0.343, P<0.001). Additionally, the severity of self-injury was positively correlated with self-stigma (r=0.289, P<0.001), neuroticism (r=0.226, P<0.01), extraversion-introversion (r=0.232, P< 0.01), and psychoticism (r=0.233, P<0.01). (4) Family history of psychiatric disorders and self-stigma can partially predict the frequency of self-injury among outpatient adolescents (F = 3.344, p<0.01); self-stigma is a risk factor for the severity of self-injury in this population (OR = 1.05, 95% CI: 1.01-1.10).

**Conclusions:**

Outpatient adolescents with a family history of psychiatric disorders and higher levels of self-stigma exhibit greater frequencies of self-injury; furthermore, those with elevated self-stigma show higher severity of self-injury.

## 1 Introduction

Non-suicidal self-injury refers to the deliberate, repetitive harm to the individual’s own body using methods not socially or culturally accepted, such as cutting, biting, scratching, or burning (1). This behavior is performed intentionally and directly (2) without the intent to end the individual’s life, typically serving as a means of emotional regulation (3). Since non-suicidal self-injury (NSSI) was included in the fifth edition of the Diagnostic and Statistical Manual of Mental Disorders (DSM-5), it has attracted widespread attention (4). Numerous studies have demonstrated that non-suicidal self-injury is particularly prevalent among adolescents and has become a significant public health issue affecting their mental health (5, 6). Survey data indicate that the prevalence of NSSI among Chinese adolescents ranges from 24.8% to 27.4% (7, 8). In community samples from western countries, the prevalence of NSSI behavior among adolescents is 16.9% (9). This indicates that NSSI among adolescents has a high prevalence, seriously threatening their physical and mental health as well as social functioning.

According to the self-injury stigma theoretical framework proposed by Staniland, stigma is categorized into four interrelated dimensions: public stigma, anticipated stigma, enacted stigma, and self-stigma (10). This theoretical framework emphasizes that experiencing stigma may not only increase an individual’s psychological burden but also reduce their willingness to seek help, delay recovery, and in some cases, exacerbate self-injurious behavior (15). Given the multiple sources of stigma, this study focuses primarily on the negative evaluations perceived by adolescents due to their emotional distress and self-injurious behaviors, rather than stigma arising solely from outpatient treatment labeling. Therefore, guided by this theoretical framework, the present study thoroughly investigates the impact of public stigma and self-stigma on the frequency and severity of self-injury among adolescents.

Public stigma refers to the negative stereotypes, discrimination, and prejudices held by the general public toward individuals with mental disorders (10). In this study, public stigma refers to the negative perceptions, discrimination, and prejudices held by the general public toward individuals engaging in self-injury. Such attitudes manifest in discriminatory behaviors toward adolescents with NSSI, who in turn perceive this stigma imposed by society (11, 12). Extensive research indicates that educators and healthcare professionals often hold stigmatizing attitudes toward adolescent NSSI, perceiving self-injury as shameful behavior. This stigma can reduce adolescents’ willingness to seek help, thereby exacerbating self-injury (13, 14). Other studies surveying individuals with NSSI in college and community populations have shown that perceived public stigma is associated with indicators of NSSI severity (15). Similarly, a qualitative study found that public stigma can hinder the recovery of individuals with NSSI (16). During the recovery process, self-injury may be exacerbated due to stigmatization. Therefore, this suggests that public stigma may be positively correlated with the frequency and severity of self-injury among adolescents.

Self-stigmatization refers to the internalization of public stigma by individuals, who then apply these negative beliefs to themselves (17). In this study, self-stigma related to adolescent self-injury refers to the psychological process in which adolescents internalize society’s negative stereotypes and prejudices toward self-injury, leading to feelings of shame, self-blame, self-deprecation, and low self-esteem. Research indicates that, among adolescents with NSSI, this internalized self-stigma can inhibit the disclosure of self-injury (18), increase internalized shame (19), and further exacerbate self-injurious behavior. A study assessing both implicit and explicit attitudes of self-stigma in individuals with NSSI found that participants with lower levels of explicit bias were more likely to have engaged in more severe NSSI behaviors (20). Based on the self-injury stigma framework proposed by Staniland, it can be inferred that self-stigma functions through the internalization of external negative evaluations, generating self-blame and shame. This process influences the psychological state and behavioral patterns of individuals who self-injure, potentially reducing their willingness to seek support, increasing psychological stress, and ultimately exacerbating the severity of self-injury (10). Therefore, based on theoretical perspectives and existing research, these findings collectively suggest that self-stigma may be correlated with higher frequency and greater severity of self-injury among adolescents.

Adolescents are in the pubertal stage, during which they undergo rapid changes in physiological, psychological, and social adaptation (21). Personality traits, as one of the core factors in adolescent psychological development, play a significant role in the occurrence and progression of self-injurious behavior and therefore should not be overlooked (22). This study targeted adolescents attending psychiatric outpatient clinics for self-injurious behavior, aiming to comprehensively understand the occurrence of self-injury in this population and further explore the relationships between public stigma, self-stigma, and both the frequency and severity of adolescent self-injury. The objective is to provide empirical evidence to reduce the stigmatization of adolescent self-injury. Based on the above literature, we propose the following hypotheses:1) Public stigma is positively correlated with the frequency and severity of self-injury among adolescents attending psychiatric outpatient clinics; 2) Self-stigma is positively correlated with the frequency and severity of self-injury in this population.

## 2 Materials and methods

### 2.1 Study design and participants

Using a convenience sampling method, 143 adolescents aged 12–18 who attended psychiatric outpatient clinics between February and October 2024 and met the DSM-5 diagnostic criteria for non-suicidal self-injury were selected. After excluding 13 invalid questionnaires, a total of 130 adolescents with NSSI were included in the final analysis. Inclusion criteria: 1) Adolescents seeking treatment for self-injury at psychiatric specialty hospitals typically may present with mental health conditions such as depressive disorders, anxiety disorders, adjustment disorders, or conduct disorders; however, the participants included in this study did not meet the relevant diagnostic criteria according to the DSM-5; 2) aged 12–18 years, regardless of gender; 3) meeting the DSM-5 diagnostic criteria for non-suicidal self-injury. Exclusion criteria: 1) individuals with severe neurological or physical illnesses; 2) individuals with reading, comprehension, or communication difficulties that prevent understanding of the questionnaires; 3) individuals with language communication disorders; those diagnosed with organic mental disorders, intellectual disability, pervasive developmental disorders, attention-deficit/hyperactivity disorder, or schizophrenia were excluded.

The assessment of NSSI behaviors was conducted according to the DSM-5 research criteria (4): the individual must have engaged in five or more NSSI episodes in the past year, with at least one episode occurring in a month; these behaviors are primarily intended to relieve negative emotions or cope with psychological distress, rather than being motivated by suicidal intent; the behaviors may result in significant psychological distress or impairment in social functioning, but should not be attributable to schizophrenia, substance use disorders, or other psychiatric or medical conditions. The diagnosis for all participants was jointly conducted by at least two psychiatrists with associate chief physician qualifications or higher, based on the DSM-5 criteria for NSSI and integrating clinical interviews and medical record reviews for comprehensive assessment. This study did not employ structured diagnostic interview tools, primarily because the outpatient setting limited the feasibility of systematically implementing such instruments and research resources were constrained. Consequently, the diagnostic process relied on the clinical judgment of qualified psychiatrists.

Informed consent was obtained from all participants in this study. Participants were informed of the confidentiality principles, all data were anonymized, and the data were used solely for scientific research purposes. For adolescents under the age of 16, informed consent was obtained from their legal guardians, and a written consent form was signed. This study was approved by the Institutional Review Committee of Beijing Huilongguan Hospital(2024-51-Science).

### 2.2 Measuring instruments

#### 2.2.1 Socio-demographic characteristics

The general information questionnaire, which was self-designed, mainly includes sociodemographic data: gender (male or female), only child status, left-behind child status, residence (rural or urban), and educational level (0–9 years, >10 years); general situation data: presence of self-injury among close relatives, family history of psychiatric disorders, and parental marital status (divorced or not divorced).

#### 2.2.2 Frequency and severity of self-injury

This study utilized the Adolescent Self-Harm Questionnaire revised by Feng Yu (23), which was developed based on Graze’s conceptualization of self-harming behaviors (24) and involved a localized adaptation of the Adolescent Self-Injury Questionnaire originally compiled by Deng Ying in terms of content expression (25). The questionnaire consists of 18 items, divided into assessments of the frequency of self-injurious behaviors and the severity of bodily harm. The level of self-injury is assessed by calculating the product of the frequency of self-injury and the average severity of bodily harm. The frequency of self-injury is scored on a 4-point scale from 0 to 3, representing 0 times, 1 time, 2–4 times, and 5 or more times, respectively. The severity of self-injury is scored on a 5-point scale from 0 to 4, representing none, mild, moderate, severe, and extreme, respectively. The frequency and severity scales of this questionnaire can be used separately (26). In this study, the score for self-injury frequency was calculated as the sum of all items assessing the frequency of self-injury. Based on the median score of self-injury severity, participants were divided into two groups: low severity (<14.5 points) and high severity (≥14.5 points). This questionnaire is suitable for Chinese adolescent populations and demonstrates high reliability (Cronbach’s α = 0.85).

#### 2.2.3 Self-stigma

This study utilized the Internalized Stigma of Mental Illness Scale, revised by Li Qiang and Tan Hua based on the Self-Stigma Scale developed by Ritsher, to assess self-stigma in adolescents (27, 28). The scale consists of 29 items and uses a 4-point Likert scoring system (1 to 4 points represent strongly disagree, disagree, agree, and strongly agree, respectively) (29). The scale is divided into five dimensions: self-alienation, experiences of discrimination, perceived incompetence, escape from reality, and social avoidance. The total score is the sum of all dimensions; a higher total score indicates a greater level of self-stigma perceived by the individual. The Chinese revised version demonstrated strong psychometric properties (Cronbach’s α = 0.92).

#### 2.2.4 Public stigma

Yin Huifang translated Link’s Perceived Devaluation-Discrimination Scale into Chinese and conducted reliability and validity testing (30, 31). This study adopted the Chinese version of the Perceived Devaluation-Discrimination Scale to assess individuals’ perceived public stigma. The scale contains 12 items, of which 6 are reverse-scored. Each item is rated on a 4-point Likert scale, with 1 to 4 representing strongly disagree, disagree, agree, and strongly agree, respectively. The total score is the sum of all items; a higher total score indicates a higher level of perceived public stigma. The Chinese version of the scale demonstrated acceptable internal consistency (Cronbach’s α = 0.70).

#### 2.2.5 Personality traits

The Eysenck Personality Questionnaire Junior Version revised by Gong Yaoxian was used to assess adolescents’ personality traits (32). It includes four factors: extraversion, neuroticism, psychoticism, and social desirability, with a total of 88 items scored dichotomously as 0 or 1, and standardized T-scores calculated based on normative data. The scale demonstrated an internal consistency reliability of 0.84. The Chinese revised version demonstrated high reliability (Cronbach’s α = 0.84).

### 2.3 Survey methods

First, patients were evaluated by a psychiatrist with associate chief physician qualifications, and if they met the DSM-5 diagnostic criteria for NSSI, they proceeded to complete the questionnaire assessments (4). The questionnaire assessments were conducted under the supervision of a physician qualified as a psychotherapist. Prior to the assessments, the purpose, content, procedure, rights and privacy, benefits, and risks of the study were explained to the participants and their guardians. After obtaining consent from the participants and their guardians, informed consent forms were signed. Additionally, the assessments were conducted in a quiet and comfortable psychological testing room to maximize scientific rigor, with the total testing time ranging from 15 to 20 minutes. To enhance the reliability of the assessments, validity-check items, such as “ How many seasons are there in a year? “, were included in the questionnaires. After data collection, our researchers excluded questionnaires that were incomplete, fabricated, or had identical answers throughout. A total of 130 valid questionnaires were obtained.

### 2.4 Statistical analysis

This study used G*Power 3.1 software to estimate the sample size based on multiple linear regression analysis (fixed model, R² deviation from zero) (33). Based on commonly used standards in psychological research (34), the effect size was set at f²=0.15 (medium effect), the significance level at α=0.05, the statistical power at 1−β=0.85, and the model was assumed to include five independent variables. The calculation results indicated that the minimum sample size required to complete this study was 102.

Statistical analyses were conducted using SPSS 27.0 and Rx64 4.0.3. Tests for common method bias, multicollinearity diagnostics, descriptive statistics, group comparisons, Pearson correlation analysis, multiple regression analysis, and binary logistic regression analysis were performed using SPSS 27.0 software. Calculations involving biserial correlation analyses were conducted in R, as it provides dedicated functions for accurately computing biserial correlations, suitable for assessing the association between dichotomous and continuous variables (35).

Harman’s single-factor test was used to assess common method bias (36). Categorical data were described using frequencies (n) and percentages (%), while continuous data were described using means (M) and standard deviations (SD). To assess the distribution characteristics of continuous variables, normality tests were conducted for each variable, and the data were generally approximately normally distributed. Group comparisons were conducted using chi-square (χ²) tests and independent samples t-tests. Pearson correlation analysis was used to examine the relationships among continuous variables, including self-stigma, public stigma, personality traits, and the frequency of self-injury. Biserial correlation analysis was employed to examine the relationships between artificially dichotomized variables (self-injury severity) and continuous variables (self-stigma, public stigma, and personality traits). Since self-injury severity is an artificially dichotomized variable, biserial correlation was selected, as Pearson correlation is not appropriate.

Prior to conducting regression analyses, multicollinearity among independent variables was assessed using the variance inflation factor (VIF). The results showed that the tolerance values for all models were <1, with VIFs ranging from 1.04 to 1.58, indicating no serious multicollinearity issues. Finally, multiple regression analysis was used to explore the effects of self-stigma, public stigma, and personality traits on self-injury frequency; binary logistic regression analysis was conducted to examine their effects on self-injury severity. The significance level was set at 0.05.

## 3 Results

### 3.1 Common method bias test

In this study, Harman’s single-factor test was employed to assess the presence of common method bias (36). All items were included in an exploratory factor analysis. The results indicated that 21 factors had eigenvalues greater than 1, with the first common factor accounting for 20.58% of the variance, which is below the critical threshold of 40% (36). Therefore, it can be inferred that there is no significant common method bias in this study.

### 3.2 Descriptive analysis of stigmatization and self-injurious behaviors among outpatient adolescents

Among the 130 outpatient adolescents, the total score for self-injury frequency was 19.61 ± 10.26. A total of 67 adolescents (51.5%) exhibited low levels of self-injury, while 63 adolescents (48.5%) demonstrated high levels of self-injury. The total score for self-stigma was 75.13 ± 14.28, while the total score for public stigma was 32.35 ± 6.25. See **Table 1**.

**Table 1:** Basic characteristics of stigmatization and self-injurious behaviors among the adolescent sample.

Variables	M ± SD/N(%)
Self-injurious behavior	
Frequency of self-injurious behavior	19.61 ± 10.26
Severity of self-injurious behavior	
Mild-severity self-injury (1-14.5 points)	67(51.5%)
High-severity self-injury (≥14.5points)	63(48.5%)
Stigmatization	
self-stigma	75.13 ± 14.28
Public stigma	32.35 ± 6.25

### 3.3 Comparison of self-injurious behaviors among outpatient adolescents with different characteristics

The results of the chi-square test indicated that parental divorce status (χ² = 5.898, P = 0.015) and family history of psychiatric disorders (χ² = 9.922, P = 0.003) differed significantly between the high and low self-injury groups. The results of the independent samples t-test showed that adolescents with a family history of psychiatric disorders (t = -2.637, P = 0.009) had higher scores for the frequency of self-injury compared to those without such a history. See **Table 2**.

**Table 2:** Comparison of self-injury severity and frequency among adolescents with different characteristics.

Variables	Mild-severity self-injury N(%)	High-severity self-injury N(%)	*χ^2^*	*P*	Frequency of self-injury M ± SD	*t*	*P*
Gender			0.727	0.394		-0.001	1.000
Female	57(85.1)	50(79.4)			19.61 ± 10.20		
Male	10(14.9)	13(20.6)			19.61 ± 10.77		
Hometown			0.036	0.849		-0.032	0.974
Rural areas	18(26.9)	16(25.4)			19.56 ± 10.50		
Urban areas	49(73.1)	47(74.6)			19.63 ± 10.23		
Single-child			0.535	0.465		-0.727	0.469
No	33(49.3)	27(42.9)			18.90 ± 10.00		
Yes	34(50.7)	36(57.1)			20.21 ± 10.51		
Left-behind children			2.404	0.121		-0.608	0.544
No	65(97.0)	57(90.5)			19.47 ± 10.14		
Yes	2(3.0)	6(9.5)			21.75 ± 12.56		
Years of education			1.642	0.200		1.836	0.069
0-9	34(50.7)	39(61.9)			21.05 ± 10.95		
>10	33(49.3)	24(38.1)			17.75 ± 9.06		
Parental divorce			5.898	0.015		-0.934	0.352
No	60(89.6)	46(73.0)			19.21 ± 10.37		
Yes	7(10.4)	17(27.0)			21.38 ± 9.77		
Family history of self-injury			2.772	0.096		-1.216	0.226
No	61(91.0)	51(81.0)			19.17 ± 10.17		
Yes	6(9.0)	12(19.0)			22.33 ± 10.73		
Family history of psychiatric disorders			9.922	0.003		-2.637	0.009
No	63(94.0)	47(74.6)			18.62 ± 10.11		
Yes	4(6.0)	16(25.4)			25.05 ± 9.59		

### 3.4 Correlation analysis of self-stigma, public stigma, personality traits, and the frequency and severity of self-injury among outpatient adolescents.

Pearson correlation analysis showed that the frequency of self-injury scores among outpatient adolescents was positively correlated with self-stigma (r = 0.343, P < 0.001). Biserial correlation analysis revealed that the severity of self-injury among outpatient adolescents was positively correlated with self-stigma (r = 0.287, P < 0.001), neuroticism (r = 0.226, P < 0.01), extraversion-introversion (r = 0.232, P < 0.01), and psychoticism (r = 0.233, P < 0.01). Further details can be found in **Table 3**.

**Table 3:** Correlation analysis of self-stigma, public stigma, personality traits, and frequency and severity of self-injury.

Variables	1	2	3	4	5	6	7	8	9	10	11	12
1.self-stigma	1											
2.Self-alienation	0.915 ^a^	1										
3.Experience of discrimination	0.901 ^a^	0.756 ^a^	1									
4.perceived incompetence	0.796 ^a^	0.668 ^a^	0.679 ^a^	1								
5.Escape from reality	0.581 ^a^	0.402 ^a^	0.463 ^a^	0.368 ^a^	1							
6.Social avoidance	0.794 ^a^	0.662 ^a^	0.653 ^a^	0.508 ^a^	0.423 ^a^	1						
7.Public stigma	0.571 ^a^	0.519 ^a^	0.482 ^a^	0.464 ^a^	0.329 ^a^	0.500 ^a^	1					
8.Neuroticism	0.009	-0.008	0.101	-0.002	-0.020	-0.074	-0.110	1				
9.Extraversion-introversion	0.025	0.024	0.011	0.060	-0.141	0.108	0.051	-0.547 ^a^	1			
10.Psychoticism	0.021	0.019	0.011	0.058	-0.147	0.104	0.049	-0.174	0.999 ^a^	1		
11.Frequency of self-injury	0.343 ^a^	0.331 ^a^	0.294 ^a^	0.256 ^a^	0.061	0.378 ^a^	0.139	-0.130	0.115	0.116	1	
12.Severity of self-injury	0.287 ^a^	0.247 ^b^	0.310 ^a^	0.228 ^b^	0.025	0.287 ^a^	0.095	0.226 ^b^	0.232 ^b^	0.233 ^b^	0.849 ^a^	1

^a^ *P* < 0.001; ^b^ *P* < 0.01.

### 3.5 Regression analysis of outpatient adolescents with self-injury frequency as the outcome variable

Using self-injury frequency as the dependent variable and gender, parental divorce history, family history of psychiatric disorders, self-stigma, public stigma, neuroticism, extraversion-introversion, and psychoticism as independent variables, a multiple regression analysis was performed using the entry method. Family history of psychiatric disorders and self-stigma entered the regression model with significant effects, indicating that these two variables jointly explain 22.5% of the variance in self-injury frequency (F = 3.344, p < 0.01). The final regression equation can be expressed as Y = 6.434*family history of psychiatric disorders + 0.235*self-stigma + 11.839. See **Table 4**.

**Table 4:** Regression analysis with frequency of self-injury as the outcome variable.

Regression equation		Overall goodness-of-fit index			Significance of regression coefficients			
Outcome variable	Predictor variable	*R*	*R ^2^*	*F*	*B*	*β*	*t*	*P*
Frequency of self-injury		0.475	0.225	3.344 ^b^				
	Gender				-1.169	-0.042	-0.423	0.673
	Parental divorce				1.331	0.050	0.525	0.601
	Family history of self-injury				6.434	0.222	2.315	0.023
	self-stigma				0.235	0.322	2.905	0.005
	Public stigma				-0.342	-0.210	-1.876	0.064
	Neuroticism				-0.032	-0.041	-0.364	0.717
	Extraversion-introversion				0.176	0.148	1.280	0.204
	Psychoticism				0.118	0.161	1.718	0.089

b *P* < 0.01.

### 3.6 Multivariate logistic regression analysis of outpatient adolescents with self-injury severity as the outcome variable

Using self-injury severity as the dependent variable, gender, parental divorce history, and family history of psychiatric disorders in outpatient adolescents were controlled as confounding factors, and other variables were selected using the forward selection method to conduct multivariate logistic regression analysis. The results indicated that self-stigma (OR = 1.05, 95% CI: 1.01-1.10) is a risk factor for self-injury severity among outpatient adolescents. See **Table 5**.

**Table 5:** Binary logistic regression analysis with severity of self-injury as the outcome variable.

Logistic regression equation							OR95 % CI	
Outcome variable	Predictor variable	*B*	*B-SD*	*Wald*	*P*	*Or*	*Lower limit*	*Upper limit*
Severity of self-injury	Gender	-0.232	0.694	0.112	0.738	0.793	0.204	3.087
	Parental divorce	0.614	0.591	1.080	0.299	1.848	0.580	5.883
	Family history of self-injury	1.246	0.684	3.315	0.069	3.477	0.909	13.298
	self-stigma	0.049	0.021	5.516	0.019	1.051	1.008	1.095
	Public stigma	-0.054	0.044	1.474	0.225	0.948	0.869	1.034
	Neuroticism	0.002	0.021	0.007	0.932	1.002	0.962	1.043
	Extraversion-introversion	0.029	0.034	0.736	0.391	1.030	0.963	1.101
	Psychoticism	0.023	0.017	1.921	0.166	1.023	0.991	1.057

## 4 Discussion

In China, a 2020 survey conducted by Han Azhu found that the detection rate of self-injury among adolescents was 27.4% (7). A 2024 survey by Zhu found that the detection rate of NSSI among hospitalized adolescents was 32.28% (37). In the present study, the detection rate of high self-injury severity among adolescents was 48.5%, with an average self-injury frequency score of 19.61, which is higher than that reported in previous studies (7–9, 37). This may be attributed to the unique characteristics of the sample selected in the present study. In previous studies, most adolescents with NSSI were recruited from community or school settings, whereas the present study focused on adolescents seeking care at psychiatric outpatient clinics. According to the experiential avoidance model of self-injury, when individuals are unable to tolerate intense negative emotions, they may engage in self-injurious behaviors driven by experiential avoidance motives to alleviate immediate negative emotions and achieve short-term emotional relief (38). The adolescents selected for this study sought clinical treatment due to self-injurious behaviors and often experience persistent interpersonal and academic stress in daily life. In response to stressful events, they tend to experience heightened negative emotions (39). However, these adolescents exhibit insufficient emotion regulation abilities, leading them to use self-injury more frequently as a coping strategy for stress (40). Therefore, compared to adolescents in community and school samples, those seeking treatment at specialized psychiatric clinics tend to exhibit higher levels and frequencies of self-injury. This indicates that adolescents seeking treatment at psychiatric clinics experience more severe psychological distress and suggests that they may more frequently engage in NSSI as a short-term emotion avoidance strategy.

The results of this study indicate that a family history of psychiatric disorders positively predicts the frequency of self-injury among outpatient adolescents; specifically, those with a family history have higher self-injury frequency scores. This is consistent with previous research (41). Previous studies have shown that adolescents with a family history of psychiatric disorders may have increased emotional vulnerability through genetic mechanisms, and may also be adversely affected by parenting styles and family atmosphere within the household, impacting their mental health (42, 43). Moreover, adolescents with a family history of psychiatric disorders tend to have weaker self-regulation abilities when facing stressful events compared to those without such a history, and receive relatively less familial support, which further diminishes their capacity to cope with negative events (44). This dual disadvantage may lead adolescents with a family history of psychiatric disorders to be more likely to use self-injury as a means of emotional regulation and coping with stress, thereby increasing the frequency of self-injurious behaviors. Therefore, clinical practice should pay close attention to adolescents with a family history of psychiatric disorders, assess their emotional regulation abilities and the effectiveness of their family support systems, and implement targeted interventions to reduce their risk of self-injury.

The results of this study indicate that adolescents’ extraversion, psychoticism, and neuroticism are all positively correlated with the severity of self-injurious behaviors. This finding is partially consistent with previous research, which has shown that adolescents with higher levels of neuroticism typically exhibit greater incidence and severity of self-injurious behaviors, potentially due to emotional instability, weaker stress-coping abilities, and more frequent experiences of negative emotions (45, 46). However, this study further found that, in addition to neuroticism, both extraversion and psychoticism are also positively correlated with the severity of self-injurious behaviors. This suggests that the influence of personality traits may be broader and more complex, with adolescents’ personality types not only affecting their emotional responses but also potentially exerting direct or indirect effects on the manifestation of self-injurious behaviors.

The results of this study indicate that the level of self-stigma perceived by adolescents can positively predict both the frequency and severity of their self-injurious behaviors. Specifically, outpatient adolescents experiencing higher levels of self-stigma tend to have correspondingly higher scores in both self-injury frequency and severity. This finding supports hypothesis 1 proposed in this study and is consistent with previous research findings (47). Existing literature indicates that self-stigma causes adolescents to perceive themselves as “different from others” or “socially unacceptable,” leading to negative self-evaluations (16, 48). In this context, adolescents are more likely to engage in self-injurious behaviors as a means of regulating emotions or coping with psychological stress (49, 50). Moreover, experiences of being labeled negatively may further exacerbate feelings of helplessness, creating a vicious cycle that intensifies self-injurious behaviors. Therefore, addressing and reducing the stigma experienced by adolescents holds significant theoretical and practical implications for the prevention and intervention of self-injurious behaviors.

This study found that public stigma did not significantly predict the frequency or severity of self-injury among outpatient adolescents. This result is consistent with findings from some previous studies. A study on university students who engage in self-injury indicated that although these students generally perceive public stigma in society, this perception did not have a significant impact on their self-injurious behaviors (51). Other studies have suggested that public stigma may play a certain role in the occurrence and development of self-injurious behaviors among adolescents (52). Specifically, negative societal labeling of self-injurious behaviors may cause adolescents to feel ashamed to express psychological distress, thereby hindering their proactive help-seeking (52). This suggests that the impact mechanism of public stigma on adolescent self-injurious behavior may be complex; public stigma may indirectly influence self-injury by hindering help-seeking, and may also exhibit heterogeneous effects depending on individuals’ perceived levels of stigma. Therefore, there is currently no consensus on the specific pathways through which public stigma affects adolescent self-injurious behavior. Future research is needed to further investigate potential mediating mechanisms and moderating variables to clarify the role of public stigma in adolescent self-injury.

Previous studies have indicated that approximately 80% of hospital-treated NSSI patients have comorbid psychiatric disorders, with depressive and anxiety disorders being the most prevalent (53). This study focused on adolescents seeking psychiatric treatment who met the DSM-5 criteria for NSSI but did not fulfill the diagnostic criteria for other psychiatric disorders. To minimize the potential confounding effects of other psychiatric disorders, such as anxiety disorders, depressive disorders, conduct disorders, and adjustment disorders, strict exclusion criteria were applied, ensuring that none of the 130 adolescents included in the sample met the diagnostic criteria for other psychiatric disorders. This measure enhanced the homogeneity of the sample to some extent, allowing the findings to more accurately reflect NSSI symptoms, but it inevitably reduced ecological validity. Considering that many adolescents with NSSI in real-world settings present with multiple comorbidities, excluding these groups may underestimate the complexity of NSSI in clinical contexts. In this study, the sample was drawn from a single psychiatric outpatient clinic using convenience sampling, which may introduce selection bias and thereby limit the generalizability of the findings to adolescents in community or school settings. Although this study applied the DSM-5 criteria for NSSI, diagnoses were primarily based on clinicians’ judgment without the use of structured diagnostic interviews, which may affect diagnostic consistency and reliability.

In future research, the sample size could be increased by recruiting participants from different regions and various types of medical institutions, as well as from community or school settings, in order to enhance the external validity and generalizability of the findings. Secondly, potential psychiatric comorbidities in adolescents should be systematically assessed, and these comorbidities should be included as control or grouping variables in statistical analyses to examine the effects of public and self-stigma on NSSI behaviors. In terms of data analysis, diversified and refined statistical approaches could be employed to further explore the complex mechanisms among variables, thereby providing more robust empirical evidence for reducing the stigma associated with self-injury.

## 5 Conclusions

The data for this study were obtained from adolescents with NSSI in psychiatric outpatient settings, providing a systematic investigation of the impact of stigma on NSSI among outpatient adolescents. The study results indicate that adolescents with a family history of psychiatric disorders and higher levels of self-stigma exhibit greater self-injury frequency, and that higher self-stigma is also associated with increased self-injury severity. Therefore, future interventions targeting self-injurious behaviors among outpatient adolescents should pay special attention to those with a family history of psychiatric disorders and elevated levels of self-stigma. For these high-risk adolescents, more targeted interventions should be developed and implemented, such as conducting family history screenings, enhancing mental health education, reducing stigma associated with psychiatric disorders, and providing personalized psychological support services to reduce their risk of self-injury.

## Data Availability

The raw data supporting the conclusions of this article will be made available by the authors, without undue reservation.
